# SARS-CoV-2 evolution among patients with immunosuppression in a nosocomial cluster of a Japanese medical center during the Delta (AY.29 sublineage) surge

**DOI:** 10.3389/fmicb.2023.944369

**Published:** 2023-02-09

**Authors:** Yoshie Hosaka, Yan Yan, Toshio Naito, Rieko Oyama, Koji Tsuchiya, Norio Yamamoto, Shuko Nojiri, Satoshi Hori, Kazuhisa Takahashi, Yoko Tabe

**Affiliations:** ^1^Department of Clinical Laboratory Medicine, Juntendo University Faculty of Medicine, Tokyo, Japan; ^2^Department of General Medicine, Juntendo University Faculty of Medicine, Tokyo, Japan; ^3^Department of Research Support Utilizing Bioresource Bank, Juntendo University Faculty of Medicine, Tokyo, Japan; ^4^Department of Microbiology, Tokai University School of Medicine, Hiratsuka, Kanagawa, Japan; ^5^Medical Technology Innovation Center, Juntendo University Faculty of Medicine, Tokyo, Japan; ^6^Infection Control Unit, Juntendo University Hospital, Tokyo, Japan; ^7^Department of Respiratory Medicine, Juntendo University Faculty of Medicine, Tokyo, Japan

**Keywords:** SARS-CoV-2, Delta variant, AY.29, immunosuppression, mutation, genome sequencing, nosocomial cluster

## Abstract

**Background:**

Previous studies have shown that patients with immunosuppression tend to have longer-lasting SARS-CoV-2 infections and a number of mutations were observed during the infection period. However, these studies were, in general, conducted longitudinally. Mutation evolution among groups of patients with immunosuppression have not been well studied, especially among Asian populations.

**Methods:**

Our study targeted a nosocomial cluster of SARS-CoV-2 infection in a Japanese medical center during Delta surge (AY.29 sublineage), involving ward nurses and inpatients. Whole-genome sequencing analyses were performed to examine mutation changes. Haplotype and minor variant analyses were furtherly performed to detect the mutations on the viral genomes in detail. In addition, sequences of the first wild-type strain hCoV-19/Wuhan/WIV04/2019 and AY.29 wild-type strain hCoV-19/Japan/TKYK15779/2021 were used as references to assess the phylogenetical development of this cluster.

**Results:**

A total of 6 nurses and 14 inpatients were identified as a nosocomial cluster from September 14 through 28, 2021. All were Delta variant (AY.29 sublineage) positive. 92.9% of infected patients (13 out of 14) were either cancer patients and/or receiving immunosuppressive or steroid treatments. Compared to AY.29 wild type, a total of 12 mutations were found in the 20 cases. Haplotype analysis found one index group of eight cases with F274F (N) mutation and 10 other haplotypes with one to three additional mutations. Furthermore, we found that cases with more than three minor variants were all cancer patients under immunosuppressive treatments. The phylogenetical tree analysis, including 20 nosocomial cluster-associated viral genomes, the first wild-type strain and the AY.29 wild-type strain as references, indicated the mutation development of the AY.29 virus in this cluster.

**Conclusion:**

Our study of a nosocomial SARS-CoV-2 cluster highlights mutation acquisition during transmission. More importantly, it provided new evidence emphasizing the need to further improve infection control measures to prevent nosocomial infection among immunosuppressed patients.

## Introduction

SARS-CoV-2, the causative agent of the COVID-19 pandemic, is a positive-sense single-stranded RNA virus, known to acquire new mutations at each cycle of genome replication due to the error-prone nature of the viral RNA-dependent polymerase complex (Drake, 1993; Kim et al., 2020). The mutation rates of RNA viruses are generally higher than DNA viruses, and mutations lead to the selection and evolution of viral genomes (Duffy, 2018; Peck and Lauring, 2018). Since the first report of the Delta variant of SARS-CoV-2 in India in late 2020, this variant quickly became the dominant clade globally until the Omicron variant took over soon after its first report in South Africa in November 2021 (World Health Organization, 2021). Previous studies indicate that the Delta variant has been evolving, supported by evidence of patients positive for Delta harboring newly identified mutations (Baj et al., 2021).

Meanwhile, patients with immunosuppression were reported to be at risk for prolonged infection with SARS-CoV-2, along with a number of identified substitutions and deletions in genome sequences (Choi et al., 2020; Corey et al., 2021; Kemp et al., 2021). Although cases with immunosuppression were traced longitudinally and genome sequences were conducted sporadically during their infection to identify mutations, how mutations evolve among a select group of SARS-CoV-2 infected patients with immunosuppression has not been well studied, especially in Asian populations.

AY.29, a sublineage of the Delta variant, was detected and first reported in April 2021; it quickly became predominant in Japan until the end of the year when Omicron began replacing Delta. Although AY.29 was known to have Y1658Y and V1750A in ORF1ab (NSP3) mutations (EPI_ISL_2723567/EPI_ISL_2723568; Abe and Arita, 2021; Koyama et al., 2022), studies on further mutation acquisition have remained scant. With increased transmission and hospitalization rates compared to previous variants of concern (VOC), nosocomial clusters have been reported worldwide during the Delta surge, including those occurring in hospitals with strict infection control measures (Klompas et al., 2021; Lim et al., 2021). Using whole-genome sequencing analysis of infection cases, nosocomial clusters provide a natural environment for tracing and analyzing mutation emergence. As a referral academic medical center with strict infection control protocols in Japan, Juntendo University Hospital (JUH) experienced a nosocomial cluster in September 2021 during the Delta surge, including ward nurses and inpatients with existing respiratory or rheumatological/autoimmune diseases.

To clinically better understand why this cluster rapidly developed and how mutations emerged among this group of high-risk inpatients, we performed whole-genome sequencing analysis to examine mutation evolution of the infected cases in the nosocomial cluster.

## Methods and materials

### Description of hospital and baseline infection control measures

Juntendo University Hospital is a 1,051-bed academic medical center in Japan. Since the outbreak of the COVID-19 pandemic, strict infection control measures have been implemented. At the hospital, masks are universally required of all healthcare workers and patients in all facilities including outpatient clinics and wards (when patients’ conditions allow). All inpatients are nasopharyngeal or saliva polymerase chain reaction (PCR) tested at the time of admission. Visitors to wards are prohibited in general; in pediatric wards, one PCR-tested parent may be allowed to stay with the child if needed (from admission day until the discharge day without entry and exit from the ward). For healthcare workers, in addition to universal masking, face shield or eye protection is required when encountering all patients; additionally, use of N95 respirators when caring for patients with suspected or confirmed COVID-19 is mandated. Temperature checks occur daily at the workplace, with COVID-19 symptoms requiring further examination; dining with more than three non-family members outside work hours is discouraged per hospital policy. Close contacts of confirmed cases are PCR-tested and quarantined. Because of these strict infection control measures, there was not a major nosocomial cluster of COVID-19 until September 2021 in this hospital.

### Detection of nosocomial cluster

Two nurses who worked in Ward I and reported COVID-19 symptoms on September 13, 2021, were confirmed to be infected by PCR positive tests on September 14. Immediately, all healthcare workers of Ward I, including doctors, nurses and administrative staff, as well as close contacts, were screened by PCR tests and frequently tested thereafter as new cases were identified in the ward. Healthcare workers with confirmed infection were quarantined immediately after detection for a defined period (7 days after symptom onset, or 7 days in total for asymptomatic cases). For the patients of Ward I, PCR tests were conducted on close contacts of confirmed cases or having COVID-19 symptoms. For patients of Ward II on the same floor as Ward I, PCR tests were conducted if patients reported COVID-19 symptoms. All patients of confirmed infection were transferred to COVID-19 wards of the hospital immediately after detection, or into private rooms for temporary stay until COVID-19 rooms became available.

### Collection of respiratory specimens and RT-PCR

For diagnosis of SARS-CoV-2 infection, nasopharyngeal and saliva tests (both proved To have high sensitivity and specificity) were performed (Yokota et al., 2021). Nasopharyngeal swabs were performed following a standardized procedure (World Health Organization, 2006). For saliva sampling, The participants collected 1–2 mL of unstimulated saliva into a sterile 50-mL polyethylene tube. Nasopharyngeal swabs and saliva samples were submitted for RT-PCR testing within 3 h after collection (Pandit et al., 2013). RT-PCR was carried out using the 2019 novel coronavirus detection Kit (nCoV-DK; Shimadzu corporation, Kyoto, Japan). The nCoV-DK assay uses the “2019-nCoV_N1” primer and probe sequences as described by the United States CDC’s “2019-novel coronavirus real-time rRT-PCR panel primers and probes” (Centers for Disease Control and Prevention, 2020). This assay also includes internal control oligonucleotides. Specific spike protein variations (L452R, N501Y, E484K, E484Q) were detected with the VirSNiP SARS-CoV-2 mutation assays (Roche diagnostics, Rotkreuz, Switzerland) according to manufacturer instructions. Real-time PCR analysis was run on a light cycler system (Roche, California, United States).

### Next generation sequencing

Purified RNA was reverse-transcribed into cDNA using the SuperScript VILO cDNA synthesis kit (Invitrogen, Carlsbad, CA, United States), and the synthesized cDNA was amplified with the Ion AmpliSeq SARS-CoV-2 Research Panel (Thermo Fisher Scientific, Waltham, MA, United States) on the Ion GeneStudio S5 System according to manufacturer instructions. The Ion AmpliSeq SARS-CoV-2 Research Panel consists of 2 primer pools targeting 237 amplicons tiled across the SARS-CoV-2 genome, with an additional 5 primer pairs targeting human expression controls. The SARS-CoV-2 amplicons range from 125 to 275 bp in length. Amplified samples were then sequenced using Ion 530 chips (Thermo Fisher Scientific) with eight samples per chip on the Ion S5 system. The Torrent Suite 5.14.0 platform and specific plugins were used for Next-Generation Sequencing (NGS) data analysis. The COVID19AnnotateSnpEff (v.1.3.0) plugin was used for annotation of variants. SARS-CoV-2 variants with frequencies of SNP higher than 18% or indel higher than 25% can be reproducibly detected with sequencing depth. All analyzed sequences showed a base accuracy of over 96% and a base coverage over 45×. The pangolin software was used for the assignment of SARS-CoV-2 lineages. Sequencing reads were then submitted as FASTA files and deposited in the EpiCoV database of Global Initiative on Sharing Avian Influenza Data (GISAID) (Shu and McCauley, 2017). Amino acid substitutions in the sequenced viruses were analyzed by GISAID during the registration of the viral genomes, while information was collected from the EpiCoV database. Analysis of PANGO lineage was performed based on v.3.1.15.

Samples were processed, sequenced and analyzed according to the following schedule: Case 1 to Case 16: September 24–29, 2021; Case 17 to Case 20: October 1–6, 2021.

### Mutation analysis

Because this cluster occurred during the Delta (AY.29 sublineage) surge in the Tokyo metropolitan area, mutations of the nosocomial cluster were identified using an AY.29 strain as reference. Sequence hCoV-19/Japan/TKYK15779/2021, which was registered in April 2021 when AY.29 was first detected and reported in Japan (Koyama et al., 2022), was used. A table of mutations was prepared, with conserved mutations in all samples identified as the index type, and variables shown for other cases.

### Phylogenetic tree and haplotype network analysis

To clarify the relationship of each cluster-related virus and its relationship with AY.29, phylogenetic tree analysis was performed by using the 20 samples from the studied cluster, and the wild type SARS-CoV-2 hCoV-19/Wuhan/WIV04/2019 and the AY.29 strain hCoV-19/Japan/TKYK15779/2021 as references. These sequences were aligned with the MAFFT v7.490. Poorly aligned regions in 5′ and 3′ ends were trimmed, and the core regions were determined to be from the 55-to 29,856-nt position in the reference sequence. A Maximum Likelihood phylogenetic tree with ultrafast bootstrap support values (calculated from 1,000 replicates) was constructed by IQ-TREE 2.1.2 under the TIM2 + F nucleotide substitution model, which was selected by the ModelFinder software. The haplotype data were generated in DnaSP v6.12.03 (Rozas et al., 2017), and a median-joining network was constructed by PopART v1.7 (Leigh and Bryant, 2015).

### Detection of minor variants

Variant callers were performed with the parameters: minimum allele frequency was set to indel = 0.05; snp = 0.05; mnp = 0.05; gen_min_alt_allele_freq = 0.025; and gen_min_indel_alt_allele_freq = 0.025. Variations were annotated to the reference genome SARS-CoV-2 strain Wuhan-Hu-1 (accession number: NC_045512.1) using SARS CoV-2 annotate SnpEff. The resulting alignments were visualized to examine false positive with the Integrated Genomics Viewer (IGV) v2.15.4 (Robinson et al., 2011). Identified mutations in the cluster cases were compared to the mutations in AY.29 wild type against Wuhan-Hu-1. The different nucleotide and amino acid sequences between cluster-associated viruses and AY.29 wild type were summarized.

## Results

From Sep 14 through Sep 28, 2021, a total of 20 nurses and patients in Ward I were confirmed to be infected with SARS-CoV-2. All were Delta variant positive. Characteristics of the 20 cases are shown in Table 1. The case numbers were assigned chronologically by the PCR confirmation date. Among them, 6 were nurses (age range: 23 to 50) working in Ward I, including 5 in Team A and 1 in Team B; 5 were fully vaccinated with COVID-19 mRNA vaccines (two doses) and 1 partially vaccinated (1 dose). None needed medical care; 2 were asymptomatic while 4 had light symptoms such as runny nose, fatigue, cough, joint pain, or sore throat.

**Table 1:** Characteristics of infected nurses and patients in the nosocomial cluster, September 14 through September 28, 2021.

									Nurse	Patient				
	Haplotype^a^	Sex	Age	PCR-confirmed date	SARS-CoV-2 variant	CT value of PCR test	COVID-19 symptoms at confirmation of infection	Vaccination	Team A/B	Room type at symptom onset of infection^b^	Diagnosis at admission	Cancer (Yes/No)	Under immunosuppressive treatment (Yes/No)	Under steroid treatment (Yes/No)
**Case 1**	Type 2 (index type)	F	30	2021/9/14	Delta (AY.29)	21.62	Runny nose; joint pain	Fully (2 doses)	Team A	--	--	--	--	--
**Case 2**	Type 2	F	25	2021/9/14	AY.29	25.37	Fatigue	Partially (1 dose)	Team A	--	--	--	--	--
**Case 3**	Type 11	F	50	2021/9/15	AY.29	17.31	Runny nose	Fully	Team A (Post breast cancer surgery)	--	--	--	--	--
**Case 4**	Type 2	F	73	2021/9/15	AY.29	11.20	Fever	Not yet	--	4-bed room E	Polymyalgia rheumatica	No	No	Yes (prednisolone, 40 mg)
**Case 5**	Type 4	M	60	2021/9/16	AY.29	30.48	Fever	N/A	--	4-bed room F	Small cell lung cancer	Yes	Yes (carboplatin, etoposide)	Yes (dexamethasone, 6.6 mg)
**Case 6**	Type 9	F	31	2021/9/16	AY.29	20.43	Fever; coughs	Not yet	--	4-bed room E	Behçet’s disease	No	No	No
**Case 7**	Type 7	M	52	2021/9/16	AY.29	24.35	Asymptomatic	Not yet	--	4-bed room D	Polymyositis	No	Yes (Neoral 300 mg)	Yes (prednisolone, 5 mg)
**Case 8**	Type 7	F	28	2021/9/17	AY.29	29.63	Asymptomatic	Fully	Team A	--	--	--	--	--
**Case 9**	Type 2	M	73	2021/9/17	AY.29	18.39	Asymptomatic	Fully	--	4-bed room D	Non-small cell lung cancer	Yes	No	No
**Case 10**	Type 2	F	67	2021/9/17	AY.29	20.94	Asymptomatic	Fully	--	4-bed room B	Systemic sclerosis	No	No	Yes (methylprednisolone, 6.5 mg)
**Case 11**	Type 1	F	77	2021/9/17	AY.29	22.84	Asymptomatic	Fully	--	4-bed room B	Lung adenocarcinoma	Yes	Yes (methotrexate, 4 mg)	Yes (prednisolone, 5 mg)
**Case 12**	Type 5	F	23	2021/9/18	AY.29	25.91	Asymptomatic	Fully	Team B	--	--	--	--	--
**Case 13**	Type 3	M	49	2021/9/18	AY.29	30.79	Fever; coughs	Not yet	--	4-bed room D	Adult Still’s disease	No	No	Yes (prednisolone, 60 mg)
**Case 14**	Type 10	F	31	2021/9/19	AY.29	27.93	Fever	Not yet	--	4-bed room A	Mixed connective tissue disease	No	Yes (Tacrolimus, 2.4 ng/ml)	Yes (prednisolone, 30 mg)
**Case 15**	Type 8	F	46	2021/9/21	AY.29	13.33	Coughs	Not yet	--	4-bed room B	Systemic lupus erythematosus	No	No	Yes (prednisolone, 40 mg)
**Case 16**	Type 2	F	28	2021/9/21	AY.29	22.45	Sore throat	Fully	Team A	--	--	--	--	--
**Case 17**	Type 2	F	68	2021/9/23	AY.29	29.87	Fever; coughs	Partially	--	4-bed room E	Lung adenocarcinoma	Yes	No	Yes (prednisolone, 2.5 mg)
**Case 18**	Type 6	M	68	2021/9/24	AY.29	21.55	Fever	Fully	--	Private room 7	Lung adenocarcinoma	Yes	No	No
**Case 19**	Type 2	M	70	2021/9/25	AY.29	28.63	Fever	N/A	--	4-bed room C	Infiltrative thymoma	Yes	Yes (tegafur/gimeracil/ oteracil)	No
**Case 20**	Type 6	M	18	2021/9/28	AY.29	25.30	Fever; coughs; runny nose	Not yet	--	Ward II of the same floor	Mediastinal germ cell tumor	Yes	Yes (bleomycin)	No

^a^Types were defined based on haplotype-based analysis. ^b^Except for case 20, room types here indicate rooms in Ward I.

Regarding the 14 infected patients (age range: 18 to 77), 7 had rheumatology/autoimmune diseases; 7 had respiratory diseases. 92.9% of these infected patients (13 out of 14) were either cancer patients and/or receiving immunosuppressive or steroid treatments. Specifically, 6 out of 7 patients with rheumatology/autoimmune diseases were under immunosuppressive and/or steroid treatment; for the 7 patients with respiratory disease, all had cancer and 5 were either under immunosuppressive and/or steroid treatment. Only 4 (out of 14) infected patients had been fully vaccinated; 1 was partially vaccinated; 9 were either unvaccinated or without available vaccination record. A total of 3 senior patients, all aged more than 70 and with severe existing conditions, died after identification of the Delta variant infection.

**Figure 1:**
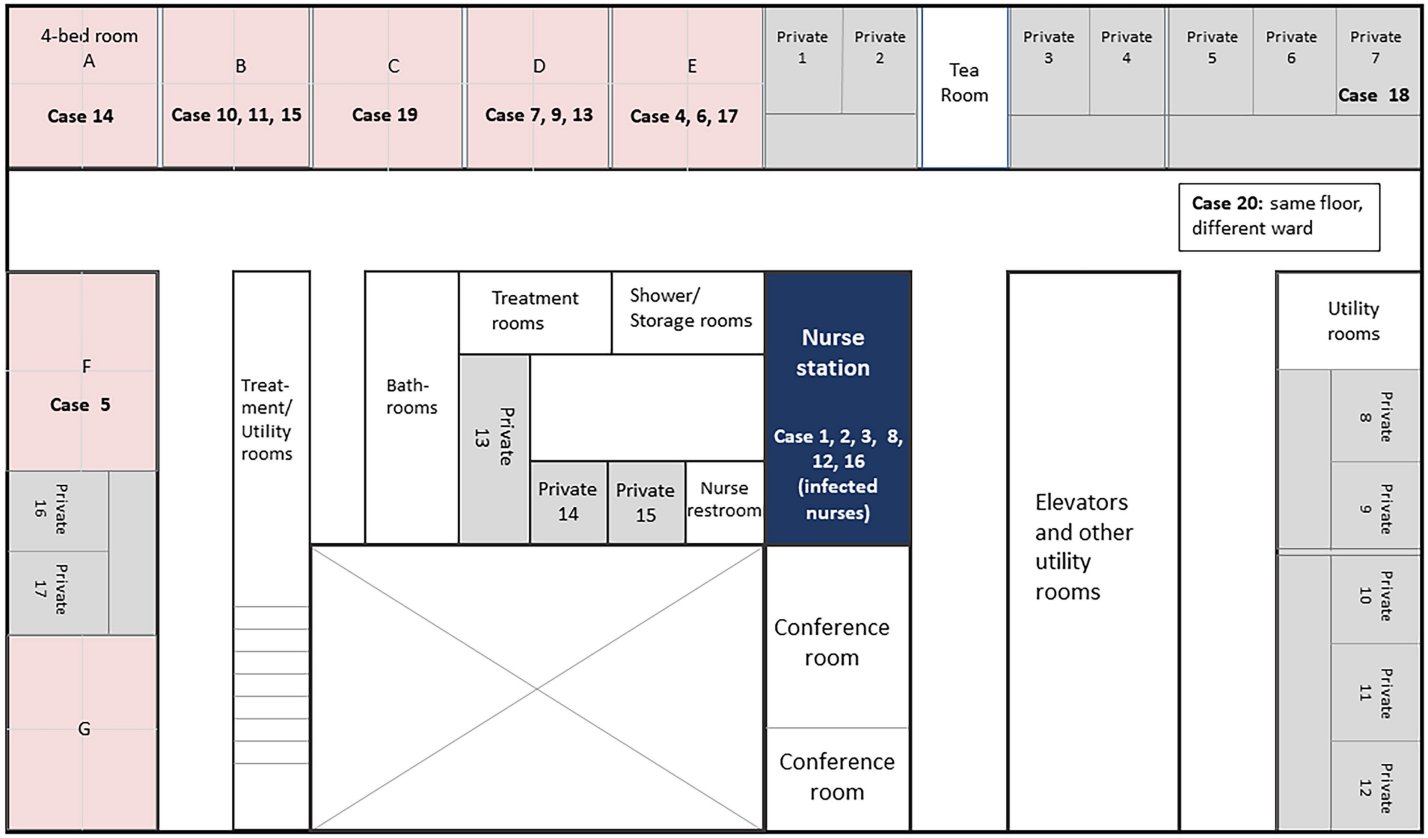
Floor map of Ward I in Juntendo University Hospital with cases at the onset of SARS-CoV-2 infection. Room numbers indicate patient location at the onset of SARS-CoV-2 infection (showing various symptoms including fever, cough, and/or running nose). After diagnosis of SARS-CoV-2, patients were transferred either to COVID-19 wards directly or temporarily to the private rooms within the same ward before transferring to COVID-19 wards.

Ward I of this medical center consists of 7 rooms with 4 beds each, 14 regular private rooms, and 3 private rooms adjacent to the nurse station for patients who may need immediate attention. Layout of these rooms is shown in Figure 1. Except for case 18, all patients were found to be in shared rooms A, B, C, D, E, or F at symptom onset of SARS-CoV-2 infection. These patients were then transferred to either COVID-19 wards directly or temporarily to the private rooms inside the ward before moving to the COVID-19 wards.

**Figure 2:**
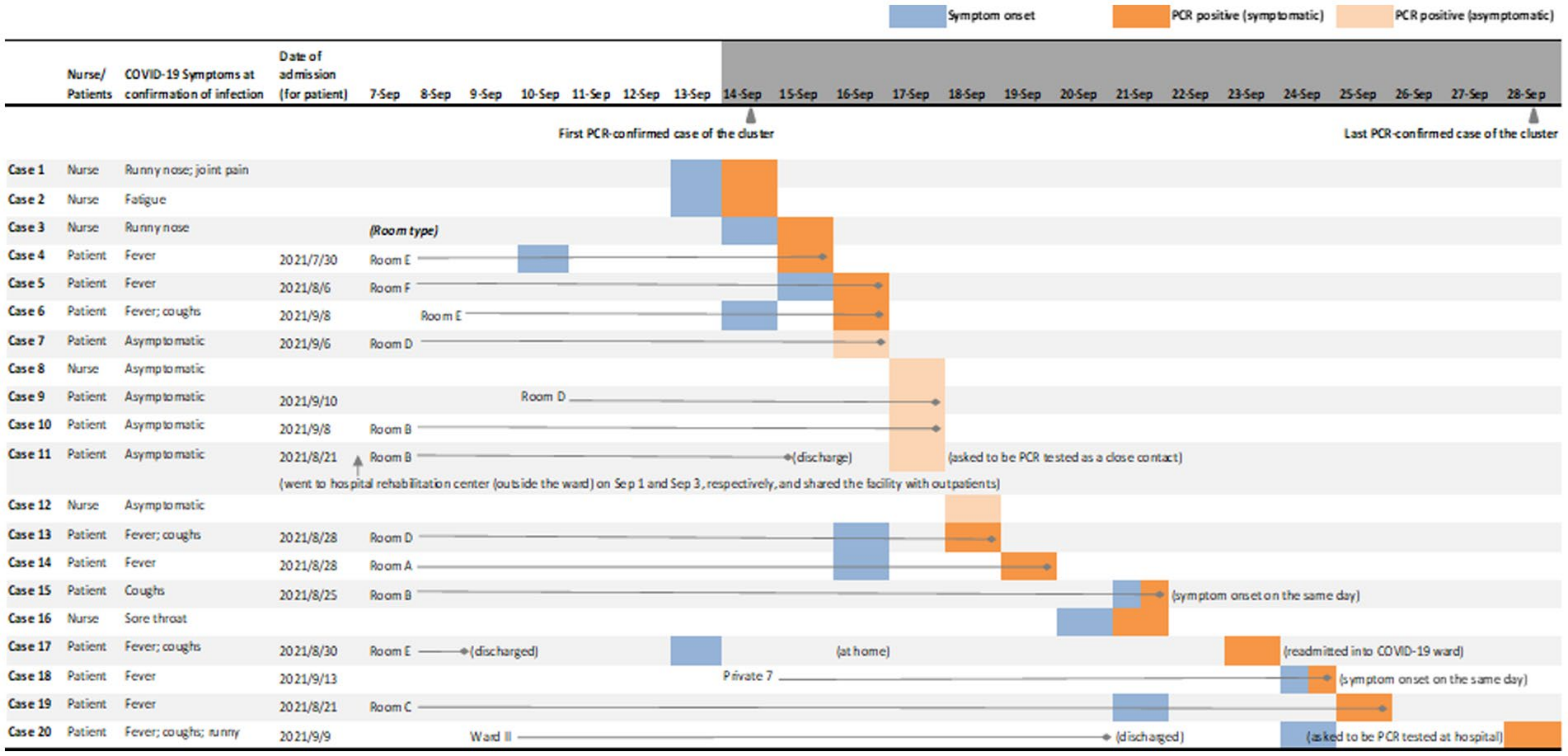
Symptoms and PCR-confirmed detection of SARS-CoV-2 infection cases in the nosocomial cluster. This figure shows the PCR-confirmed infected patients’ room type (for inpatients at the timepoint of 1 week prior to the first PCR-confirmed case as September 7, 2021, or their admission date if admitted thereafter), the date of symptom onset and the date of PCR positive test; for infected nurses, date of symptom onset and the date of PCR positive test are shown. Type Room A to F are four-bed shared rooms; along with Private 7, these rooms are all located in Ward I. Case 20 stayed in Ward II of the same floor until discharged on September 21, reported symptoms on September 24, and tested positive on September 28. No other patients in Ward II were detected during the period of the nosocomial cluster. Patients might be transferred during their stay in Ward I due to reasons such as care requirements (for instance, increased proximity to the nurse station); these internal transfers are not shown in this figure. After PCR-confirmed detection, patients were transferred either to COVID-19 wards directly, or to private rooms inside Ward I for temporary stay until transferring to COVID-19 wards; all infected nurses were quarantined immediately after a PCR positive test. Immediately after the first two cases were detected on September 14, 2021, all healthcare workers of Ward I, including doctors, nurses and administrative staff, as well as close contacts, were screened by PCR tests and frequently tested thereafter as new cases were identified in the ward.

Figure 2 illustrates the dates of PCR detection and any accompanying symptoms. The first two nurses were PCR-confirmed on September 14, 2021. After PCR testing for all healthcare workers of the ward and any inpatients identified as close contacts, 6 cases were then detected asymptomatically, including Case 11 who was identified as a close contact. Case 11 was a 77-year-old female patient of lung adenocarcinoma who left Ward I for rehabilitation during her stay, was discharged, but then asked to return for PCR testing and readmitted after positive detection. Case 20 was identified lastly on September 28, after he was discharged from Ward II (the same floor as Ward I), developed COVID-19 symptoms at home and returned to be PCR tested.

**Figure 3:**
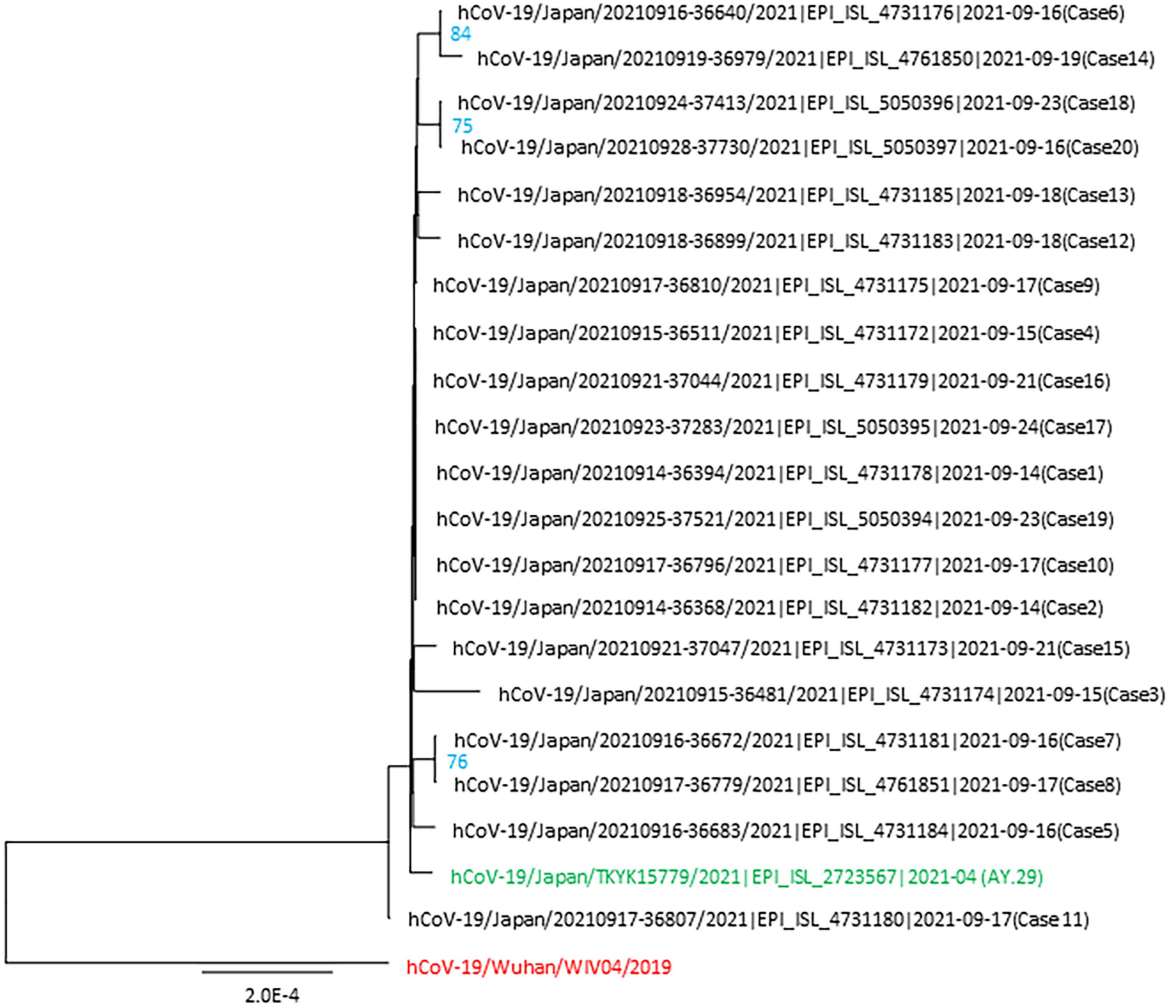
Phylogenetic tree of the SARS-CoV-2 genomes identified in the nosocomial cluster. This tree includes 20 viral genomes associated with the cluster, the first wild-type strain hCoV-19/Wuhan/WIV04/2019 and AY.29 wild-type strain hCoV-19/Japan/TKYK15779/2021 was used as the references. Each genome from the cluster is indicated with the virus name, accession ID of GISAID, and the case number. The ultrafast bootstrap support values of more than 70% were shown in the constructed tree.

The complete SARS-CoV-2 genomes showed the signatures of the Delta variant (AY.29 sublineage), which was the clade primarily circulating in Tokyo’s metropolitan area (Tani-Sassa et al., 2021; Tsuchiya et al., 2022). Phylogenetic tree analysis included 20 samples from the studied cluster, wild type SARS-CoV-2 and AY.29 (hCoV-19/Japan/TKYK15779/2021) as references. The consensus tree, generated from 1,000 replicates, is shown in Figure 3. The log-likelihood of this tree was −40836.33. The cluster-associated viruses were shown to be very similar but some of them had different nucleotide sequences, which indicated the mutation development of the AY.29 virus in this cluster.

**Figure 4:**
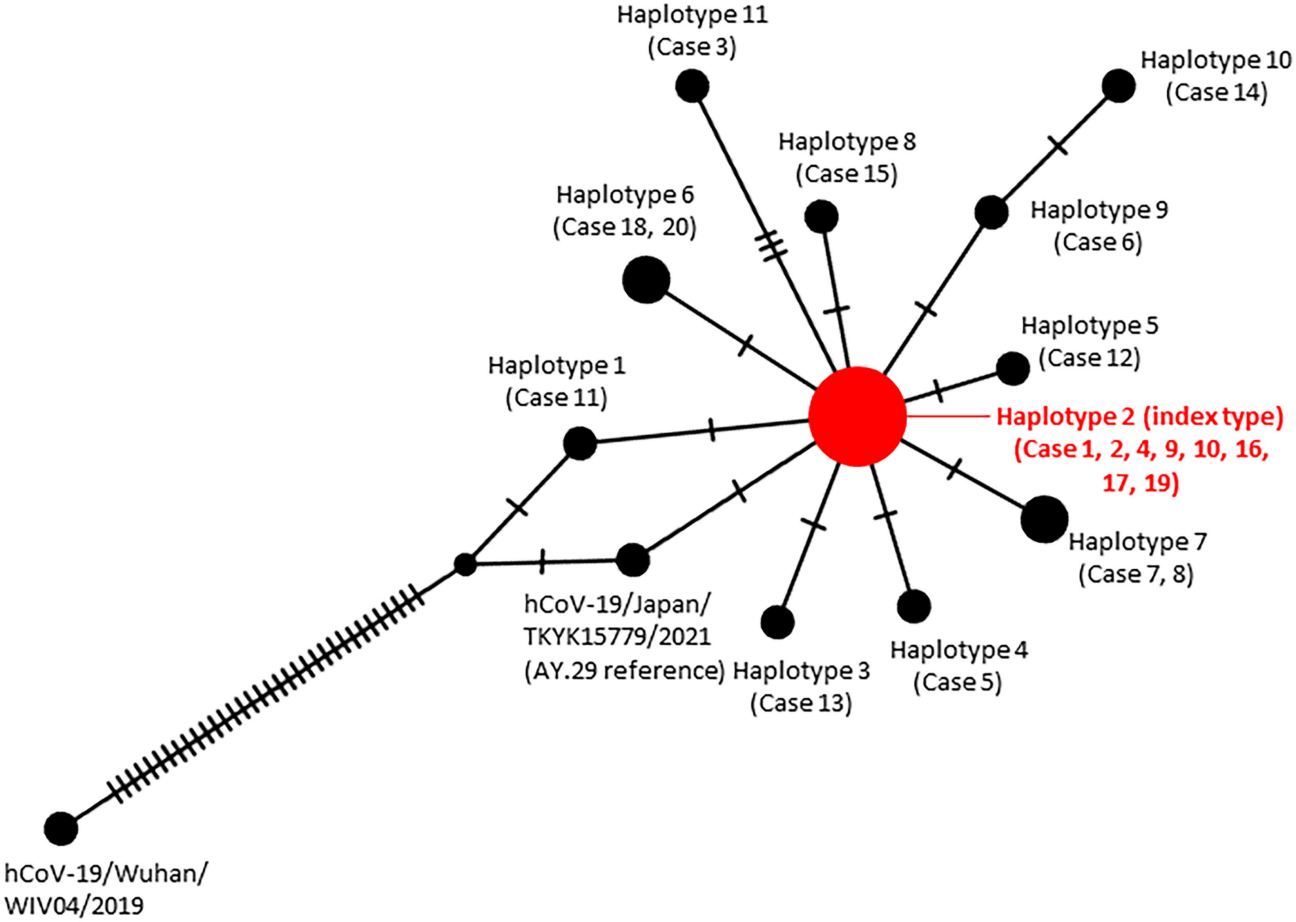
Haplotype network analysis of the SARS-CoV-2 genomes identified in the nosocomial cluster. The size of each node is proportional to the number of samples that belong to that haplotype.

Mutation analysis of these 20 sequences revealed 12 mutations compared to AY.29 (hCoV-19/Japan/TKYK15779/2021). In addition, haplotype analysis found that 8 cases shared a common mutation of F274F (N) among the samples, therefore defined as the index type. However, case 11 was found lacking G142D in S compared to the AY.29 wild type. All other cases had acquired one to three additional mutations, either non-synonymous or synonymous. Most of the genetic changes identified were located in the ORF1ab gene, followed by the S gene **(**Table 2; Figure 4**)**.

**Table 2:** Additional mutations compared to the AY.29 wild-type reference sequence.^a^

Haplotype 1	Haplotype 2 (index type)	Haplotype 3	Haplotype 4	Haplotype 5	Haplotype 6	Haplotype 7	Haplotype 8	Haplotype 9	Haplotype 10	Haplotype 11
Case 11	Case 1; 2; 4; 9; 10; 16; 17; 19	Case 13	Case 5	Case 12	Case 18; 20	Case 7; 8	Case 15	Case 6	Case 14	Case 3
F274F (N)	F274F (N)	F274F (N)	F274F (N)	F274F (N)	F274F (N)	F274F (N)	F274F (N)	F274F (N)	F274F (N)	F274F (N)
(without G142D (S) compared to AY.29 wild type reference)		G618G (ORF1ab)	S3099L (ORF1ab)	I2501T (ORF1ab)	L3935L (ORF1ab)	A65V (ORF8)	H2659H (ORF1ab)	Y489Y (S)	Y489Y (S)	I3944T (ORF1ab)
									L140L (ORF1ab)	A4577T (ORF1ab)
										T1006I (S)

a AY.29 was first reported in Japan in April 2021, and the sequence of hCoV-19/Japan/TKYK15779/2021|EPI_ISL_2723567|2021–04 was selected as the reference of the AY.29 wild type.

Table 2 presents these additional mutations, in order of the cases’ genetic distance to the index type. Specifically, case 13 obtained a mutation G618G in ORF1ab (NSP2) compared with the index type; case 5 had S3099L in ORF1ab (NSP4); case 12 obtained a mutation I2501T in ORF1ab (NSP3). Case 18 and 20 acquired a synonymous mutation L3935L in ORF1ab (NSP7); case 7 and 8 acquired an amino acid substitution A65V in ORF8. A mutation found in case 15 was a synonymous H2659H in ORF1ab (NSP3). While case 6 had a synonymous mutation Y489Y in the S gene, case 14 additionally had L140L in ORF1ab (NSP1). Case 3 possessed I3944T in ORF1ab (NSP8), A4577T in ORF1ab (NSP12), and T1006I in Spike.

Minor variants in the cluster-related viruses compared to the AY.29 wild-type reference sequence are shown in Table 3, with nine minor variants found in Case 17, three in Case 19, and four in Case 20. These three cases were all cancer patients under immunosuppressive treatment. Regarding the transmission of the viruses with minor variants, each minor variant was found only in patients or nurses individually, but not transmitted to another host.

**Table 3:** Minor variants in the cluster-related viruses compared to the AY.29 wild-type reference sequence.^a^

Case No.	Position (Wuhan-Hu-1 numbering)	Ref	Alt	Read depth	Allele frequency	Variant type	Gene	Amino acid modification
Case 1	N/A							
Case 2	21,646	C	T	15,776	0.11500	Synonymous variant	S	Y28Y
Case 3	N/A							
Case 4	N/A							
Case 5	17,589	T	C	5,223	0.12250	Synonymous variant	ORF1ab	T5775T
	29,554	G	T	713	0.0858586	Upstream gene variant	ORF10	
Case 6	5,945	C	T	10,220	0.09500	Non-synonymous variant	ORF1ab	P1894S
Case 7	N/A							
Case 8	N/A							
Case 9	N/A							
Case 10	10,029	CCT	TCG	18,495	0.14070	Non-synonymous variant	ORF1ab	TS3255IA
Case 11	N/A							
Case 12	N/A							
Case 13	N/A							
Case 14	N/A							
Case 15	N/A							
Case 16	N/A							
Case 17	801	G	A	3,795	0.07750	Non-synonymous variant	ORF1ab	G179E
	5,313	T	C	2,921	0.10250	Non-synonymous variant	ORF1ab	L1683P
	11,201	A	TG	10,515	0.13819	Frameshift_variant	ORF1ab	T3646fs
	18,268	GA	G	4,050	0.08500	Frameshift_variant	ORF1ab	E6003fs
	19,036	G	A	2,593	0.07000	Non-synonymous variant	ORF1ab	G6258S
	22,776	A	C	3,991	0.07500	Non-synonymous variant	S	D405A
	24,458	CTTAGCTCCAAT	C	2,462	0.10579	Frameshift variant	S	S967fs
	26,111	C	T	11,006	0.15750	Non-synonymous variant	ORF3a	P240L
	26,885	C	T	5,202	0.15000	Synonymous variant	M	N121N
Case 18	11,074	CT	C	28,910	0.06650	Frameshift variant	ORF1ab	L3606fs
Case 19	23,255	T	G	11,328	0.13000	Non-synonymous variant	S	F565V
	26,681	C	T	23,345	0.14250	Synonymous variant	M	F53F
	27,945	C	T	31,822	0.08500	Stop gained	ORF8	Q18stop
Case 20	1,115	A	T	8,563	0.07500	Non-synonymous variant	ORF1ab	I284F
	6,539	C	T	13,520	0.06750	Non-synonymous variant	ORF1ab	H2092Y
	20,080	T	C	5,930	0.07500	Non-synonymous variant	ORF1ab	S6606P
	28,898	AGAATGGCTGGCAATGGCGGTGATGCTGCTCTTGCTT	A	11,782	0.08549	Disruptive inframe deletion	N	R209-L221delinsMet

a SARS-CoV-2 strain Wuhan-Hu-1 (accession number: NC_045512.1) was used as the reference genome for mapping reads. Identified mutations in the cluster cases were compared to the mutations in AY.29 wild type against Wuhan-Hu-1. The different nucleotide and amino acid sequences between cluster-associated viruses and AY.29 wild type are summarized in this table.

## Discussion

In this study, we showed that SARS-CoV-2 infection spread rapidly, mostly in a single ward, having affected a total of 20 nurses and patients within 2 weeks during the Delta surge (Delta sublineage) in a Japanese medical center. The ward, with mixed inpatients of respiratory diseases and rheumatology/autoimmune diseases, shared strict infection control measures with other wards in the medical center, including a universal mask policy for medical staff and inpatients, PCR testing before admission and a no-visitor policy for all inpatients. By combining the affected cases’ epidemiological information which includes symptom onset, PCR positive tests, and records of leaving and entering the ward, along with the complete genome sequencing analysis of nasopharyngeal or saliva samples, our study suggests that this cluster possibly started with a patient (case 11; asymptomatic). Case 11 left and re-entered the ward during her stay to use the hospital’s rehabilitation facility, where she shared rooms with outpatients. The cluster might have started from this asymptomatic case and spread *via* attending nurses and patient roommates.

In addition, our study demonstrated that the SARS-CoV-2 genome can acquire one to three additional mutations within 2 weeks during the Delta surge (AY.29 sublineage). It has been shown that viruses mutate within their hosts where they develop into variants, and the number of within-host variants tends to increase over time (Jombart et al., 2011, 2014; Tonkin-Hill et al., 2021). Regarding the rapid spread of infection and acquisition of mutations in this nosocomial cluster, we offer the following two considerations.

First, droplets/aerosol transmission in shared and confined rooms, possibly due to patient conditions which physically do not permit continuous face masking, may be a risk factor for the rapid nosocomial spread of SARS-CoV-2 infections. Except for one case, all infected patients in this nosocomial cluster of Ward I were found to be in one of the 4-bed rooms at symptom onset of their infection. Previous studies have revealed that aerosol (micrometer droplets) may be a risk factor for causing and spreading COVID-19 infections, particularly with prolonged exposure in confined spaces (Yu et al., 2004; Greenhalgh et al., 2021; Lim et al., 2021). As demonstrated by supercomputing systems, small droplets can stay airborne for hours, spreading far beyond standard social distance limits, suggesting that ventilation is as important as wearing masks (Ando et al., 2022). Our hospital has been implementing a universal masking policy, with exceptions only to those with severe conditions and ordered by doctors to not mask. Some of the patients in this cluster, especially these with severe respiratory diseases, are likely unable to continually mask. Thus, droplets/aerosol transmission is considered a risk factor for the spread of infections among inpatients in these shared rooms, suggesting the importance of ventilation, including not only air-conditioning but also air purifiers, especially in dated facilities (Morawska and Milton, 2020).

Second, cancer patients and/or patients receiving immunosuppressive or steroid treatment, are higher risk for COVID-19, and may become the hosts for rapid mutation evolution. Previous studies revealed that cancer patients are more vulnerable to SARS-CoV-2 with higher rates of hospitalization and death (Dai et al., 2020; Kuderer et al., 2020; Luo et al., 2020; Nayak et al., 2021). In addition, a previous study with 585 cancer patients found that first-time infected persons with solid tumors developed lower neutralizing antibodies against the Delta variant (Fendler et al., 2021; Mahase, 2021). Another study of 152 double-vaccinated patients hospitalized due to COVID-19 found that 40% were immunosuppressed, including those under chronic corticosteroid treatment, chemotherapy/antimetabolite treatment and anti-CD20 treatment (Brosh-Nissimov et al., 2021). In addition, the rapid evolutionary rate in immunocompromised patients has been reported previously (Choudhary et al., 2021). In this nosocomial study, involving 6 nurses and 14 patients (13 out of 14 were either cancer patients and/or receiving immunosuppressive or steroid treatments), we found that the case with the most mutations (three additional mutations compared to the index type) was a nurse of post-breast cancer surgery (case 3); furthermore, we found that cases with more than three minor variants were all cancer patients under immunosuppressive treatments. Although with a limited number of samples, our study demonstrated that cancer patients/survivors and/or patients under immunosuppressive treatments can become hosts for fast SARS-CoV-2 virus spread and evolvement.

### Limitations

There are number of limitations worth addressing. First, this is a single nosocomial cluster study during Delta (AY.29 sublineage) surge in Tokyo with a limited number of samples, without intention to fully elucidate the mechanism of Delta variant’s evolution. Second, the small sample size of the nosocomial cases did not allow us to identify a significant association between vaccination and the difference in mutation acquisition frequencies, although it has been reported that the vaccination is inversely correlated to the mutation frequency of SARS-CoV-2 Delta variants (Yeh and Contreras, 2021).

## Conclusion

Our analysis of emerging mutations in a nosocomial COVID-19 cluster highlights mutation acquisition during transmission, demonstrating rapid mutations of the Delta variant (AY.29 sublineage) within 2 weeks, especially among patients with rheumatology/autoimmune diseases, lung cancer and other respiratory diseases. More importantly, because these patients are at higher risk for becoming hosts for rapid mutations, our study provides new evidence emphasizing the need to further improve infection control measures to prevent nosocomial clusters among patients with immunosuppression, even in hospitals with already strict protocols.

## Data availability statement

The whole genome sequenced in this study can be found in online repositories. The name of the repository and accession numbers can be found at: http://getentry.ddbj.nig.ac.jp/, with accession numbers LC752087–LC752106 for the studied nosocomial cases 1–20.

## Ethics statement

This study complied with all relevant national regulations and institutional policies and was conducted in accordance with the tenets of the Declaration of Helsinki. It was approved by the Institutional Review Board (IRB) at Juntendo University Hospital, Japan (IRB #20–036). Informed consent from individual patients was waived because all samples were de-identified in line with the Declaration of Helsinki.

## Author contributions

TN: full access to all the data in the study and takes responsibility for the integrity of the data and the accuracy of the data analysis. YH, NY, YT, RO, KT, and TN: acquisition, analysis, or interpretation of data. YY, NY, and YT: draft of the manuscript. YH, SN, and YY: statistical analysis. YT, TN, and RO: administrative, technical, or material support. SH and KT: supervision. All authors: concept and design, critical revision of the manuscript for important intellectual content, and meet the ICMJE authorship criteria.

## Funding

This work was supported by Japan Agency for Medical Research and Development (AMED) (grant number JP20fk0108472).

## Conflict of interest

The authors declare that the research was conducted in the absence of any commercial or financial relationships that could be construed as a potential conflict of interest.

## Publisher’s note

All claims expressed in this article are solely those of the authors and do not necessarily represent those of their affiliated organizations, or those of the publisher, the editors and the reviewers. Any product that may be evaluated in this article, or claim that may be made by its manufacturer, is not guaranteed or endorsed by the publisher.
