# Corrigendum: SARS-CoV-2 evolution among patients with immunosuppression in a nosocomial cluster of a Japanese medical center during the Delta (AY.29 sublineage) surge

**DOI:** 10.3389/fmicb.2024.1478638

**Published:** 2024-08-23

**Authors:** Yoshie Hosaka, Yan Yan, Toshio Naito, Rieko Oyama, Koji Tsuchiya, Norio Yamamoto, Shuko Nojiri, Satoshi Hori, Kazuhisa Takahashi, Yoko Tabe

**Affiliations:** ^1^Department of Clinical Laboratory Medicine, Juntendo University Faculty of Medicine, Tokyo, Japan; ^2^Department of General Medicine, Juntendo University Faculty of Medicine, Tokyo, Japan; ^3^Department of Research Support Utilizing Bioresource Bank, Juntendo University Faculty of Medicine, Tokyo, Japan; ^4^Department of Microbiology, Tokai University School of Medicine, Hiratsuka, Kanagawa, Japan; ^5^Medical Technology Innovation Center, Juntendo University Faculty of Medicine, Tokyo, Japan; ^6^Infection Control Unit, Juntendo University Hospital, Tokyo, Japan; ^7^Department of Respiratory Medicine, Juntendo University Faculty of Medicine, Tokyo, Japan

**Keywords:** SARS-CoV-2, Delta variant, AY.29, immunosuppression, mutation, genome sequencing, nosocomial cluster

In the published article, an author name was incorrectly written as Kazuhiko Takahashi. The correct spelling is Kazuhisa Takahashi.

The authors apologize for this error and state that this does not change the scientific conclusions of the article in any way. The original article has been updated.

